# Understanding how a national clinical audit is established, maintained and sustained using the Irish Hip Fracture Database as an exemplar: a realist evaluation protocol

**DOI:** 10.12688/hrbopenres.14451.2

**Published:** 2026-09-12

**Authors:** Louise Brent, Pamela Hickey, Mary Walsh, Fidelma Fitzpatrick, Niamh A. Merriman, Elaine Byrne

**Affiliations:** 1 Division of Population Health Sciences, Royal College of Surgeons in Ireland, Dublin, Leinster, Ireland; 2 National Office of Clinical Audit, Royal College of Surgeons in Ireland, Dublin, Leinster, Ireland; 3 Department of Clinical Microbiology, Beaumont Hospital, Dublin, Leinster, Ireland; 4 Royal College of Surgeons in Ireland Department of Clinical Microbiology, Dublin, Leinster, Ireland; 5 Dublin City University School of Nursing Psychotherapy and Community Health, Dublin, Leinster, Ireland; 6 Centre for Positive Health Sciences, Royal College for Surgeons in Ireland, Dublin, Ireland

**Keywords:** Keywords: clinical audit, quality improvement, realist evaluation, healthcare

## Abstract

There is an increasing focus on clinical audit within the Irish Health System to drive quality improvement and standardise care, with many established and emerging clinical audits underway. Since the burden of data collection typically falls to busy clinical staff, understanding the impact of audit is especially crucial. However, the literature rarely explores how audits generate impact, for whom and in what context, despite the considerable effort invested in collecting this valuable data.

This study will explore how a national clinical audit is established, maintained and sustained in order to maximise impact using the Irish Hip Fracture Database (IHFD) as an exemplar. The research will be conducted through a realist evaluation lens, which aims to determine what works, for whom, how and to what extent. Realist evaluation is a theory- based approach that uses mixed-methods to explore and answer the research question. Initially programme theories will be formulated through a review of the literature, qualitative interviews, and subsequently refined with an expert panel.

The Initial Programme Theories (IPTs) will be refined through iterative rounds of consultation and context-mechanism-outcome configurations. This process will identify demi-regularities, which are semi-predictable patterns that occur often enough to be noticeable, but not universally or consistently across all contexts. These patterns will inform understanding of how the intervention (i.e. the audit) operates or impacts differently across varying contexts. Ultimately, this will inform the development of a middle-range theory to better understand developing and sustaining a clinical audit with meaningful impact. The resulting findings will have relevance and potentially inform and/or enhance the implementation and development of other audits.

Ethical approval has been granted by the Research Ethics Committee in the Royal College of Surgeons in Ireland. Results will be disseminated through peer-reviewed publication and at relevant healthcare conferences including patient and public focused conferences.

## Introduction

Clinical audit is defined as a clinically-led quality improvement process that improves patient care and outcomes by systematically measuring care against clinical standards and acting to improve care when those standards are not met. ^1, 2^ Clinical audits are the cornerstone of quality improvement in many health systems, providing assurance of care quality and driving ongoing improvements. However, relatively little is known about what mechanisms are responsible for their impact and in what context impact is achieved or not.

The Irish Hip Fracture Database is a mature audit established in the Irish Health System since 2012. It captures the care and outcomes of hip fracture patients measured against best practice clinical standards ( Figure 1). Since it’s inception, this audit has led to significant developments and improvements in care including, more direct pathways of care so patients are brought to the right hospital first, more timely throughput through the emergency department and admission to an orthopaedic ward within 4 hours, improved time to surgery, reduction in pressure ulcers, review by a geriatrician, bone health and specialist falls assessment and early mobilisation of a physiotherapist. When the IHFD commenced there were no orthogeriatric services in Ireland and up to 25% patients were being brought into hospitals without orthopaedic services. Now each of the 16 trauma services have an orthogeriatric service and 95% are brought direct to the operating hospital. The IHFD is the only condition in the HSE that has an associated financial incentive for meeting the standards of care known as the best practice tariff. This funding goes directly back into the trauma services to further improve care.

**Figure 1.**
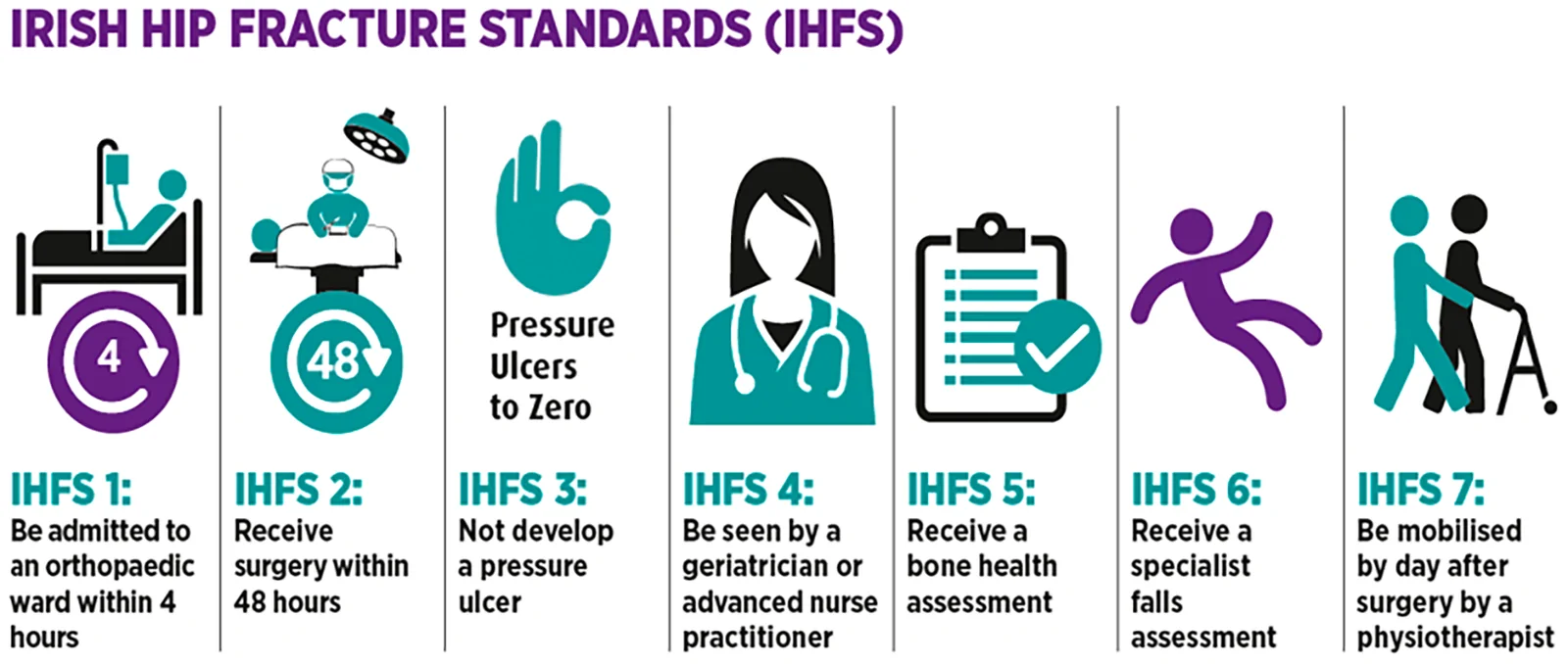
Irish Hip Fracture Standards.

There is an increasing focus on clinical audit in the Irish Health System with many established and emerging clinical audits currently in operation. As data collection is typically undertaken by clinical staff, often involving significant effort, understanding the impact of audit activity is especially important. The concept of impact in the case of the IHFD is multifactorial including anticipated, intended, unintended or emergent outcomes related to the audit activity. Some examples of impact is this context would be data quality, robust governance, compliance with the clinical standards measured by the audit, service development through organisational surveys, quality improvement activities and impact on national guidelines and policy. There is ample evidence to describe what constitutes a clinical audit and the clinical standards and outcomes that should be measured. ^3^ However, less attention has been given to investigating how clinical audit produces an impact, for whom and in what context. This gap is notable considering how much energy is expended collecting and analysing this valuable data.

A recent scoping review highlighted a paucity of literature concerning the governance of clinical audit for hip fracture. ^4^ The review described several key components integral to the governance of clinical audit including: construction of audit, content of audit, technical description, data entry process, implementation process, data quality, reporting/data dissemination, funding, management, mandatory participation and the accessibility of audit data.

The Irish Hip Fracture Database (IHFD) is being used as an exemplar for this research as it is a well-established, mature audit in operation since 2012 and underpinned by more than 12 years of nationally published data. ^5^ The IHFD has driven meaningful improvements within the Irish healthcare system, including the development of a national bypass pathway for hip fracture cases, the development of the specialty of orthogeriatrics and the establishment of Ireland’s first and currently only financial incentive for best practice, the Best Practice Tariff. To date, this has paid out over four million euros. ^6^ Given the above, the IHFD is regarded as a model from which learnings can be drawn. It therefore offers insights that may be applicable to other clinical audits or quality improvement initiatives.

The study is being conducted using a realist evaluation methodology that involves iterative development, testing, and refinement of programme theories. This study approach will adopt articulation, theory refinement, and theory verification. Initially, theory articulation will be used to develop an initial programme theory that explains how and why the intervention is expected to work, identifying the underlying mechanisms and the contextual conditions that may influence outcomes. Theory refinement will then involve testing and revising this programme theory through empirical data collection and analysis, allowing a more nuanced understanding of the context-mechanism-outcome (CMO) relationships. Finally, theory verification will assess the extent to which the refined programme theory is supported by the evidence, providing a robust explanation of what works, for whom, under what circumstances, and why. ^7, 8^ Together, these stages provide the overarching framework for the study and ensure a systematic approach to understanding programme effectiveness.

## Methods

This study is underpinned by realist evaluation methodology, which involves iterative development, testing, and refinement of programme theories. In line with this approach, the study will adopt mixed-methods using quantitative and qualitative data as detailed further below in the realist evaluation section. Participants recruited for the focus group discussions will be required to provide informed consent. Written consent will be collected either manually or electronically following each participant’s review of the participant information leaflet and consent form.

## Realist evaluation

There is an emerging use of realist evaluation within complex healthcare research. ^9^ This approach is useful for exploring a wide range of topics in healthcare including collective leadership, ^10^ the effectiveness of a rapid response model in mental state deterioration ^11^ and the evaluation of complex integrated programmes ^12^ as examples. This theory-driven approach to evaluation is grounded in scientific realism. ^8^ The seminal work by Pawson and Tilley describes realist evaluation as a mechanism to understand the effect of interventions and related contexts in which they work. ^7^ The real value of realist evaluation lies in going beyond surface-level inputs and outputs of an intervention such as a clinical audit, to uncover deeper mechanisms. This approach generates findings directly applicable to implementation and scaling. The purpose of this research method is to develop initial programme theories (IPT) which provides an explanation to how and why a clinical audit is expected to deliver impact in specific contexts. Examples of impact include data quality, robust governance, compliance with the clinical standards, service development through organisational surveys, quality improvement activities and impact on national guidelines and policy.

The steps in this realist evaluation will include constructing the initial programme theories, formulating a research protocol informed by these theories, gathering evidence to test the IPTs, configuring data around them and presenting the research findings ( Figure 2). ^8^ This will be done by exploring existing literature, using evidence from the literature to refine theories, by engaging stakeholders and experts in the field of hip fracture care and audit. Qualitative data will be collected through focus group discussions with representatives from the healthcare system involved in hip fracture care and audit and will be used iteratively to test and refine the IPT’s. These IPT’s will be described using the Context-Mechanism-Outcome (CMO) configurations. CMO configurations explore mechanisms (M) including participants’ reactions and reasonings; and their impact on a specific outcome/s (O) in specific contexts (C). ^13^ Understanding the interplay between these ‘CMO’ configurations is core to this research method e.g., (C + M = O). ( Table 1). ^7^

**Figure 2.**
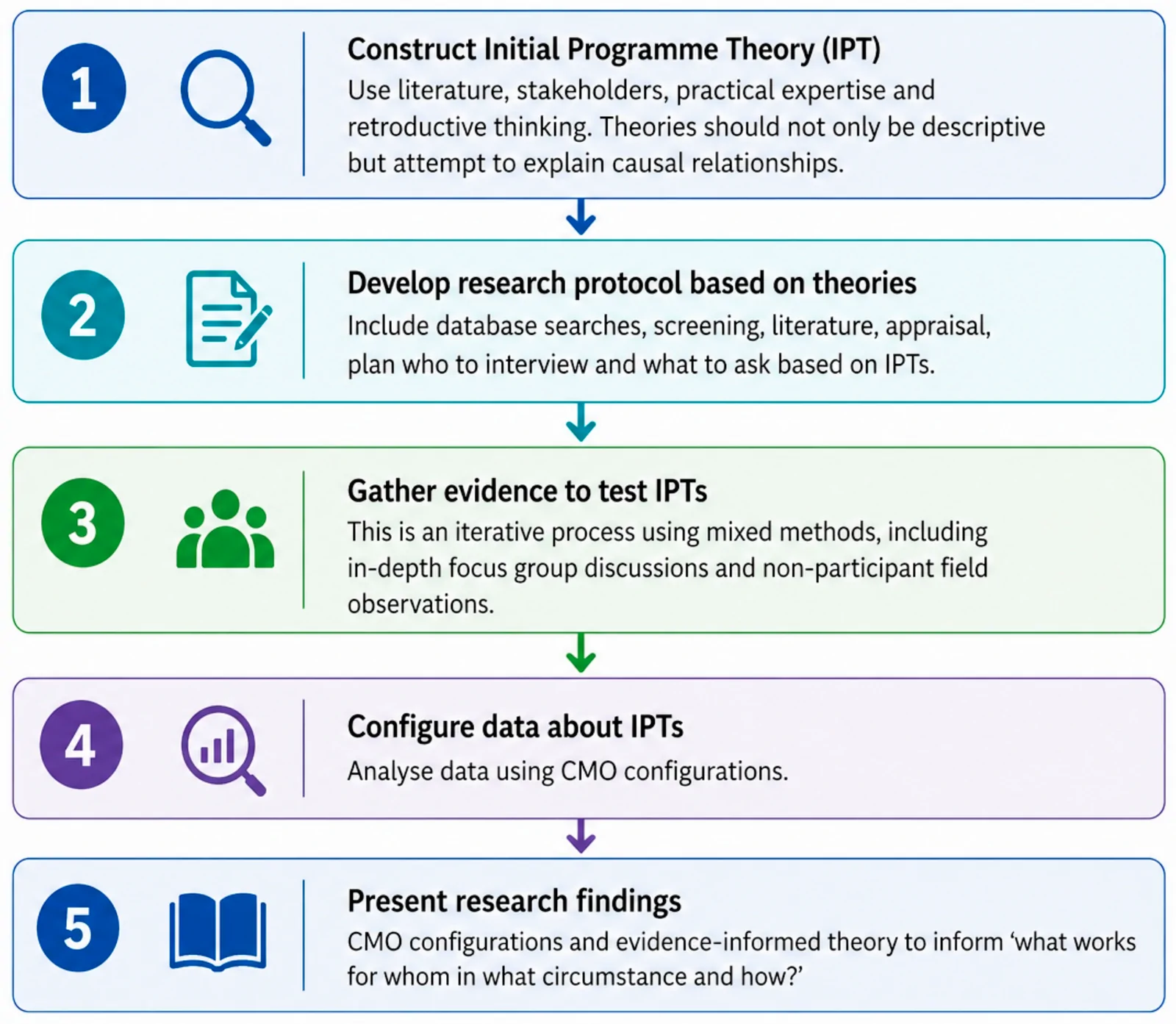
Steps of the realist evaluation process, adapted from Pawson and Tilly and Mukumbang et al. ^7, 14^

**Table 1.** Definition of context, mechanisms and outcomes used in CMO configurations in realist evaluation. ^7^

**Context**
Contexts include the relevant features of the conditions in which programmes or interventions are implemented, which are essential for understanding the functioning of mechanisms. They provide detail about the elements in the background or environment that have an impact or influence on outcomes.
**Mechanism**
Mechanisms explain what brings about changes in programmes and interventions. They are the resources within programmes or interventions that enable users to make them work. Mechanisms are the engines of explanation in realist evaluation.
**Outcome**
Outcomes include the intended and unintended effects or changes resulting from the interaction and/or activation of different mechanisms in different contexts.

The iterative refinement of the IPT’s will be completed through engagement with the research team, expert panel, focus group interviews in that sequence until information saturation is reached.

The IPTs will undergo a number of iterations as they are refined through successive rounds of data collection using different methodological approaches. A number of CMO configurations will be presented initially in relation to the early IPTs, with additional CMOs added or existing ones removed as the process evolves.

Middle Range Theories (MRTs) may emerge through this process. These are broader, transferable theories that explain social, behavioural, or organisational mechanisms (e.g., self-determination theory or governance/leadership frameworks). ^15^ MRTs help interpret and strengthen IPTs by moving beyond local assumptions and providing a theoretical lens for identifying mechanisms.

## Aims and objectives

This study aims to explore the contexts, mechanisms and outcomes, including their interactions, to provide an understanding of how the IHFD was established, maintained, evolved and sustained to have maximum impact. The IHFD will serve as an exemplar to inform other audits and patient safety initiatives.

Research question: *How does clinical audit generate impact, for whom and in what context.*

### Objectives

1. To identify and describe the contextual (C) elements of a clinical audit and describe how they interact with the various mechanisms (M) and outcomes (O).
2. To describe how the interventions worked or did not work in specific circumstances (M) and understand the impact (in terms of data quality, audit outcomes, quality improvement, governance etc), (O) based on the perceptions of staff engaged in clinical audit.

## Protocol

### Context of this research

This research will be conducted across the participating hospitals in the IHFD (N = 16) to ensure a comprehensive view of the audit’s impact. At the hospital level, the audit acts both as a local audit and as a contributor to the national audit dataset. Participation will be voluntary. The research team will deliver presentations describing the aims and objectives of the study to ensure all participants are fully informed.

### Realist evaluation cycle

The realist evaluation cycle designed by Pawson and Tilley will be used to guide this work ( Figure 3). ^7^ This cycle will be explained further in the following section.

**Figure 3.**
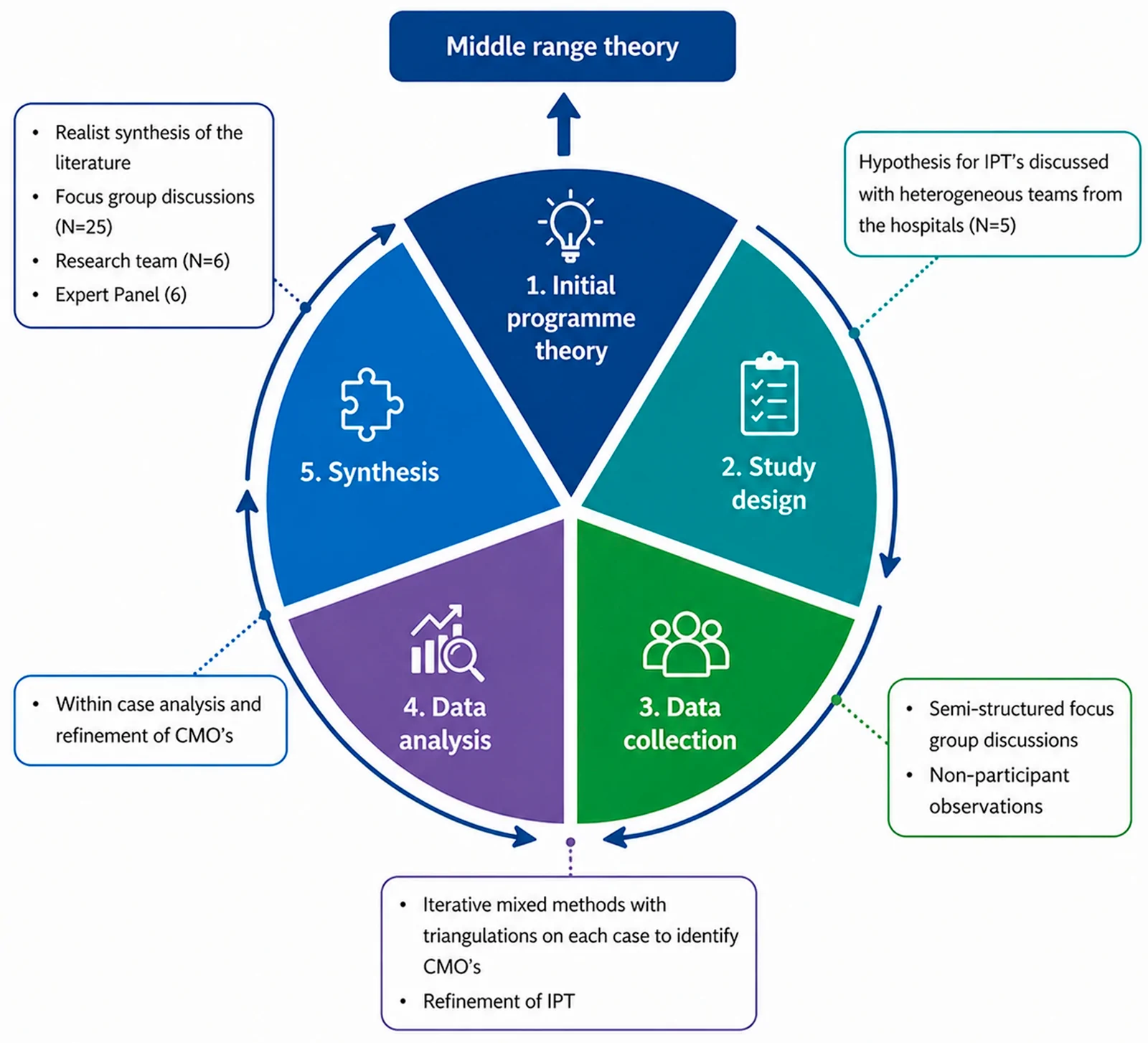
Realist evaluation cycle describing the study approach.

A number of programme theory examples (foundational or tentative theories) were formulated using ‘if’ ‘then’ statements about how the process would work and influence outcomes. Examples of these broad theories are displayed in Table 2. ^16^

**Table 2.** Programme theory examples.

Example 1: *If* healthcare professionals conducting a clinical audit focus on quality improvement rather than solely on quality assurance (C), *then* they are more likely to view the audit as a vehicle for change (M), and therefore agree and implement specific improvement actions and re audit to assess their impact on care processes (O).
Example 2: *If* the leaders of the clinical audit are champions who advocate for change (C), *then* this increases the willingness of healthcare professionals to participate and sustain that participation (M), which results in greater adoption of the improvements by individual healthcare professionals and organisations (O).

**Step 1: Formulating an initial programme theory (IPT)**

The research team will draw on a number of sources to inform the hypothesis and theory underpinning the intervention. This will involve a scoping review of the international literature and analysis and description of the evolution and impact of the IHFD. ^4, 5^

A scoping review of the literature was conducted to inform the governance of hip fracture audits. ^4^ The literature identified through this review will help inform IPT development. A template was used to extract data from papers included in the scoping review (Appendix 1). Examples of details extracted are governance components linked to specific contexts, stakeholder reasoning which could be mechanisms or outcomes and how patterns across these papers and the subsequently published papers about the IHFD will support the refinement of IPT’s by the expert panel. Further synthesis of the included papers will be undertaken to refine the IPT with the expert panel. A further analysis of the IHFD was undertaken by the research team and this will inform key attributes of the CMOs. The studies included in the scoping review described the governance of national hip fracture audits. A total of 13 papers will be included from the scoping review, representing 60% of the known national hip fracture audits globally. Eleven core areas of governance were identified through this review, and these domains will likely inform the construction of the CMOs, as they were integral to the development of many of the hip fracture audits. Subsequent papers written by the current authors describing the specific evolution of the IHFD using these 11 core governance elements and secondary uses of the IHFD data and audit will also be included. ^4, 6^

All papers identified will be assessed for their suitability for inclusion in the development of the IPTs. Data from eligible papers will be extracted using a template that will applied uniformly across all papers. Once complete, the context-mechanism-outcomes configurations will be derived and patterns across the studies will be explored.

## Study participants

### Focus group discussions

Participants will be purposively selected to represent the stakeholders who contribute to and use the audit data in participating hospitals, including staff from orthopaedics, geriatrics, emergency medicine, nursing, health and social care professions and hospital management. Each focus group will therefore bring together a diversity of roles and responsibilities, reflecting the wider stakeholder group. The initial programme theories (IPT’s) will be iteratively developed and refined early in the study, drawing from audit practice insights and the relevant literature to identify contexts, mechanisms and outcomes. A structured template will then be used to extract CMO configurations from the realist-informed focus group discussions using participants’ deliberations to further refine the IPTs before proceeding to the full realist evaluation.

### Expert panel

Senior leaders from the IHFD (including former and current clinical leads) national and international audit experts will be convened as an expert panel to further refine and collect views about the IPTs. This panel will also support the prioritisation of CMOs for subsequent testing. The IPTs will be presented to the expert panel for iterative refinement and finalised for testing. The IPTs will continue to be tested throughout successive stages of the realist evaluation process. The IHFD PPIs will be included at this stage of the process. Interviews will be conducted with individuals where necessary who have participated in the work of the IHFD at regional and national levels.

**Step 2: Study design**

The study design will be determined by the IPTs and by the most appropriate methods to test the CMOs. The research team and expert panel will be included to ensure a representative range of views and adequate coverage across the health service This phase will essentially function as a pilot phase of the realist evaluation.

The development and impact of the IHFD constitutes an intervention to improve the care and outcomes of hip fracture patients. This realist evaluation seeks to determine how this impact has been achieved. Participants will be required to have been involved with the IHFD at the hospital, level for a minimum of one year, reflecting a full cycle of the audit process culminating in the publication of a national report.

**Step 3: Data collection**

Realist evaluation is a methodology that has a neutral approach and the methods used will be determined by the IPTs. In this study, a mixed-methods design will be employed, using quantitative analysis to describe the data and evolution of the audit from data collected between 2013–2024 and three organisational surveys conducted in 2016, 2020, 2024, ^5, 17, 18^ with qualitative analysis via semi-structured focus group discussions based on realist-style questions such as “Do you think IHFD changed the way [stakeholder group] thinks or feels about [programme objective] in any way? In what ways? Can you provide examples?” Field notes will also be gathered from the non-participant sessions. Non-participant field observations are a key feature in realist evaluation and involves a three-stage funnel, starting with descriptive observation taking account of the setting, progressing to more focused observation focusing on the things that most interest the researcher and then selected observation for the thing that has been selected as being of the greatest interest. Observation is completed when theoretical saturation is reached. This process can offer more nuanced and dynamic appreciation of situations. ^19^

Most qualitative data will be generated via focus group discussions, though individual interviews may be conducted as appropriate. Focus group data will be used to test, challenge and refine the IPTs and associated CMOCs generated from the literature and other empirical sources by exploring with stakeholders how, for whom and in what circumstances the clinical audit is perceived to work, and by using their deliberations to identify additional or alternative contexts, mechanisms and outcomes. A key objective of the focus groups is to capture participants’ experiences and understandings of their involvement in, use of, and perceptions of the impact of the IHFD, in order to clarify what works (or does not work), for whom, in what contexts, and how. It is anticipated that between 25–30 participants will be recruited in total. Several focus group discussions, each ranging between 5–7 participants will be convened from these participants, to ensure a broad and diverse representation of specialties and expertise until information power is reached. ^20^ The participants will include healthcare staff from but not limited to just the following specialties, orthopaedic surgeons, geriatricians, emergency medicine, nursing, physiotherapy, occupational therapy and healthcare management. Each group will have a multidisciplinary composition to maximise the diversity of views. These participants will be sought from across the 16 participating hospitals with care given to ensure a mix of specialties and hospitals in each group. The data collection process will be iterative, with each round of focus group discussions contributing to the refinement of the CMOs and IPT’s. It is anticipated there will be approximately five rounds.

The data from the IHFD from 2013–2024 and the organisational surveys from 2016, 2020, 2024 will be used extensively throughout the study to prompt discussions and refine the CMO’s. ^5, 17, 18^

**Step 4: Data analysis**

The analysis will use a retroductive approach, which is advocated in realist research. ^21^ This approach uses both inductive and deductive logic to encourage researchers consider the scenarios posed in order to illicit the causal factors or attributes that contribute to the impact of the IHFD. As some of the factors may be not directly observable, the expertise of the researcher and their knowledge of the IHFD will play an important role in developing plausible generative explanations for how impact is produced. The findings will be developed from consensus while refining the IPTs during research team meetings. Consensus will be sought from the expert panel.

**Step 5: Synthesis**

The Analysis will be completed by drawing on multiple data sources, triangulating findings, and comparing them against the IPTs. The CMOs will then be compared with the IPTs to develop likely hypotheses regarding how various aspects of the IHFD’s work have contributed to an impact or a sustaining impact. This process will support the development of a MRT that is relevant and generalisable to other clinical audits or patient safety initiatives. As MRT is exactly that, in the middle, they serve as a launching point for subsequent theory refinement and further evaluation.

**Quality control:** The qualitative data will be managed and analysed using Nvivo software. The data will be cross-checked by two researchers before agreement is reached on refinement to the IPTs and CMOs. The RAMESES II guidelines for reporting realist evaluations will be used for this study. ^8^

### Ethics

Ethical approval has been obtained from the Research Ethics Committee in the Royal College of Surgeons in Ireland (REC202512016).

Informed written consent will be sought from all participants in advance of their participation. Participant information leaflets and consent forms will be provided and any data collected will be confidential and aggregated so that no individual is identifiable from their responses or quotes. Each participant will be given a unique identifier that only the research team will have access to ensuring anonymity for the participants in the focus group discussions.

## Discussion

Currently, relatively little is understood about how clinical audits can be sustained over time and/or how they drive measurable and continuous impact. To better understand what works (or does not), for whom, in what contexts and through what mechanisms, represents an important opportunity to leverage the learnings and experience of those involved in the IHFD. As a mature and continuously evolving national clinical audit, the IHFD has demonstrated significant and ongoing impact in Ireland, making it an ideal exemplar for this work. This research is being conducted at a time when there are calls for transparency, accountability and assurance of care within our healthcare system. The IHFD has remained resilient through many significant and challenging times, including the COVID-19 pandemic, ^22^ a national cyberattack on the Irish Health Service ^23^ and more recently, the reconfiguration of the health system into regional structures. ^24^ As such, the IHFD provides an exemplar for understanding of how clinical audits can be sustained.

This protocol outlines the basis for developing a programme theory to answer the central research question. It describes the methods guiding this realist evaluation, including the iterative and collaborative processes of refining CMOs. Realist evaluation methods will enable us to capture detail that is not routinely collected through standard clinical audit process, offering opportunities to generate new insights, and share learning with other clinical audits and patient safety initiatives.

## Dissemination

The findings from this study will be disseminated via a peer-reviewed journals targeted at key stakeholders and the results will be presented at national and international healthcare conferences. It will also be shared via the newsletter for the clinical audit organisation and network and on the IHFD website. Patient and public organisations will also be key dissemination targets. All audits actively engage with relevant PPI organisations and advocacy agencies, with PPI representative serving on all audit committees.

## Data Availability

No data are associated with this article.

## References

[1] Department of Health and Children: *Building a culture of patient safety: report of the Commission on Patient Safety and Quality Assurance.* Dublin: Stationery Office;2008. Reference Source

[2] Health Service Executive National Centre for Clinical Audit: *Nomenclature – a glossary of terms for clinical audit.* Dublin: HSE; Reference Source

[3] Health Service Executive National Centre for Clinical Audit: *A practical guide to clinical audit.* Dublin: HSE;2025. Reference Source

[4] Brent L Thies CO Fitzpatrick F : Understanding governance for a national hip fracture clinical audit: a scoping review. *Injury.* 2025;56(8):112572. 10.1016/j.injury.2025.112572 Reference Source 40627997

[5] National Office of Clinical Audit: *Irish Hip Fracture Database National Report 2024.* Dublin: NOCA;2025.

[6] Brent L Ahern E Murphy T : The development and evolution of the Irish Hip Fracture Database: a quality care initiative 2013–2024. *Archives of Osteoporosis.* 2026;21(1):96. 42342903 10.1007/s11657-026-01731-8 PMC13294304

[7] Pawson R Tilley N : *Realistic evaluation.* London: SAGE Publications;1997.

[8] Wong G Westhorp G Manzano A : RAMESES II reporting standards for realist evaluations. *BMC Med.* 2016;14:96. 27342217 10.1186/s12916-016-0643-1 PMC4920991

[9] Salter KL Kothari A : Using realist evaluation to open the black box of knowledge translation: a state-of-the-art review. *Implement. Sci.* 2014;9:115. 10.1186/s13012-014-0115-4 PMC4172789 25190100

[10] De Brún A McAuliffe E : “When there’s collective leadership, there’s the power to make changes”: a realist evaluation of a collective leadership intervention (Co-Lead) in healthcare teams. *J. Leadersh. Org. Stud.* 2023;30(2):155–172. 10.1177/15480518221144895 Reference Source

[11] Dziruni TB Hutchinson AM Keppich-Arnold S : A realist evaluation protocol: assessing the effectiveness of a rapid response team model for mental state deterioration in acute hospitals. *Front Health Serv.* 2024;4:1400060. 10.3389/frhs.2024.1400060 Reference Source PMC11284174 39076771

[12] Nurjono M Shrestha P Lee A : Realist evaluation of a complex integrated care programme: protocol for a mixed methods study. *BMJ Open.* 2018;8:e017111. 10.1136/bmjopen-2017-017111 Reference Source PMC5855239 29500199

[13] Dalkin SM Greenhalgh J Jones D : What’s in a mechanism? Development of a key concept in realist evaluation. *Implement. Sci.* 2015;10:49. 10.1186/s13012-015-0237-z PMC4408605 25885787

[14] Mukumbang FC Marchal B Van Belle S : Unearthing how, why, for whom and under what health system conditions an antiretroviral adherence club intervention works: a realist theory refining approach. *BMC Health Serv. Res.* 2018;18:315. 10.1186/s12913-018-3150-6 PMC5944119 29743067

[15] Mukumbang FC Wong G : Mechanism-based middle-range theories: using realist syntheses to reconcile specificity to context and generalizability. *Qual. Health Res.* 2025;35:1–16. 10.1177/10497323251316401 Reference Source 40073215

[16] Flynn R Schick-Makaroff K Levay A : Developing an initial programme theory to explain how patient-reported outcomes are used in healthcare settings: methodological process and lessons learned. *Int J Qual Methods.* 2020;19:1609406920916299. 10.1177/1609406920916299 Reference Source

[17] National Office of Clinical Audit: *Irish Hip Fracture Database National Report 2016.* Dublin: NOCA;2017.

[18] National Office of Clinical Audit: *Irish Hip Fracture Database National Report 2020.* Dublin: NOCA;2021.

[19] Bouncken RB Czakon W Schmitt F : Purposeful sampling and saturation in qualitative research methodologies: recommendations and review. *Rev. Manag. Sci.* 2026;20:579–615. 10.1007/s11846-025-00881-2

[20] Malterud K Siersma VD Guassora AD : Sample size in qualitative interview studies: guided by information power. *Qual. Health Res.* 2016;26(13):1753–1760. 10.1177/1049732315617444 26613970

[21] Greenhalgh T Pawson R Wong G : Retroduction in realist evaluation: the RAMESES II Project. *Health Serv Deliv Res.* 2017;5(28):1–108. 10.3310/hsdr05280

[22] Brent L Ferris H Sorensen J : Impact of COVID-19 on hip fracture care in Ireland: findings from the Irish Hip Fracture Database. *Eur Geriatr Med.* 2022;13(2):425–431. 10.1007/s41999-021-00579-9 PMC8782703 35064562

[23] Moore G Khurshid Z McDonnell T : A resilient workforce: patient safety and the workforce response to a cyber-attack on the ICT systems of the Irish health service. *BMC Health Serv. Res.* 2023;23:1112. 10.1186/s12913-023-10076-8 PMC10583305 37848947

[24] Department of Health: *Government approves HSE Health Regions Implementation Plan.* Dublin: Government of Ireland;2024. Reference Source

