# Supplementary material for: Understanding how a national clinical audit is established, maintained and sustained using the Irish Hip Fracture Database as an exemplar: a realist evaluation protocol
Source: HRB Open Res. 2026 Sep 12;9:53. Originally published 2026 May 19. [Version 2] doi: 10.12688/hrbopenres.14451.2 (PMC13650314; doi:10.12688/hrbopenres.14451.2)
Supplement: Peer review history

# Reviewer report: Reviewer response for version 2

**DOI:** 10.21956/hrbopenres.16022.r57883

**Published:** 2026-10-08

**Authors:** Lenore Anne de la Perrelle

**Affiliations:** 1 College of Nursing and Health Sciences, Flinders University, Bedford Park, Australia

The authors have addressed the need for more detailed description of the IHFD and RE methodology.

 As a protocol, the process of establishing an initial program theory to test, has been outlined well.

 The evaluation aims need to better focus on the mechanisms of establishment, maintenance and sustainment of the National audit data base and its role in improving outcomes. It would be useful to identify theory of change components such as the behaviours or conditions needed to ensure clinical audits are undertaken nationally, the governance of the audit data base to maintain and sustain national clinical audit compliance and the organisational environments that support adherence to standards and improvement of outcomes.

 The use of financial incentives is identified as a unique component and questions about those higher level mechanisms may provide useful insights to the broader issue of sustaining the national audit data base for hip fracture outcomes.

Is the study design appropriate for the research question?

Yes

Is the rationale for, and objectives of, the study clearly described?

Partly

Are sufficient details of the methods provided to allow replication by others?

Partly

Are the datasets clearly presented in a useable and accessible format?

Not applicable

Reviewer Expertise:

My expertise is in realist synthesis and evaluation of QI in health care,  mixed methods and qualitative research in aged care, palliative care and trauma-informed care

I confirm that I have read this submission and believe that I have an appropriate level of expertise to confirm that it is of an acceptable scientific standard.

## References

[1] : A realist evaluation to identify contexts and mechanisms that enabled and hindered implementation and had an effect on sustainability of a lean intervention in pediatric healthcare. *BMC Health Services Research* .2019;19(1) : 10.1186/s12913-019-4744-3 10.1186/s12913-019-4744-3 PMC688478431783853

# Reviewer report: Reviewer response for version 2

**DOI:** 10.21956/hrbopenres.16022.r57884

**Published:** 2026-09-16

**Authors:** Seán Paul Teeling, Anne Marie Keown

**Affiliations:** 1 University College Dublin, Dublin, Ireland; 2 Health Systems, University College Dublin School of Nursing Midwifery and Health Systems, Dublin, Leinster, Ireland

Thank you to the authors for their considered response to our review comments and for the revisions made to the protocol.

 We have reviewed the revised manuscript and are satisfied that our comments have been adequately addressed. In particular, the additional detail regarding the realist logic underpinning the study, the development and iterative refinement of programme theories and CMO configurations, the conceptualisation of impact, the data extraction process, and the sampling and conduct of the focus groups has strengthened the transparency and coherence of the protocol.

 The revisions to Table 2 also provide greater clarity regarding the status of the candidate programme theories and make the proposed context, mechanism and outcome relationships more visible at this stage of theory development. We recognise that these theories and their associated CMOCs will continue to be tested and refined through the realist evaluation.

 We have no further substantive comments and are happy to approve the revised protocol.

 We wish the authors well with the study.

Is the study design appropriate for the research question?

Yes

Is the rationale for, and objectives of, the study clearly described?

Partly

Are sufficient details of the methods provided to allow replication by others?

Partly

Are the datasets clearly presented in a useable and accessible format?

Not applicable

Reviewer Expertise:

My expertise is in realist review and realist evaluation, Lean and quality improvement in healthcare, person-centred process improvement, and the evaluation of complex healthcare interventions.

We confirm that we have read this submission and believe that we have an appropriate level of expertise to confirm that it is of an acceptable scientific standard.

# Reviewer report: Reviewer response for version 1

**DOI:** 10.21956/hrbopenres.15925.r55656

**Published:** 2026-06-27

**Authors:** Seán Paul Teeling, Anne Marie Keown

**Affiliations:** 1 University College Dublin, Dublin, Ireland; 2 Health Systems, University College Dublin School of Nursing Midwifery and Health Systems, Dublin, Leinster, Ireland

This is a timely and important protocol addressing how national clinical audit generates impact, for whom, and in what context. The Irish Hip Fracture Database is an appropriate and valuable exemplar, given its maturity, national reach and demonstrated contribution to improvements in hip fracture care. The study has the potential to make a useful contribution to understanding how audit activity can support improvement, sustainability and system learning.

 I agree that a realist evaluation is an appropriate design for this study. The protocol presents a valuable opportunity to move beyond describing whether clinical audit contributes to improvement, towards understanding how audit activity may generate change in different contexts and for different stakeholders. My comments are offered as suggestions and clarifications that may strengthen the transparency and coherence of the protocol, rather than as substantive changes to the proposed study design.

1. Firstly, the protocol could benefit from a slightly clearer articulation of the realist logic underpinning the work. In particular, it may be helpful for the authors to clarify how the initial program theories will be developed, tested and refined across the different phases of the study.
2. The protocol states that IPT development will be informed by the previously cited scoping review, analysis of the IHFD, audit practice insights and expert panel refinement. This is an appropriate route to IPT development in a realist evaluation. At present, the protocol states that the literature will “help inform” IPT development, that further synthesis will be undertaken, and that CMOs will be derived from eligible papers. Some additional detail on the intended analytic approach would help readers follow this process. For example, the authors might briefly clarify what template or templates will be used, what types of data will be extracted, how descriptive governance components may be considered as possible contexts, program resources, stakeholder reasoning/mechanisms or outcomes, and how patterns across the included papers and IHFD-related evidence will be identified and refined with the expert panel. This would help readers understand how the scoping review findings and IHFD analysis will contribute to realist propositions.
3. The concept of “impact” would also benefit from some clarification. In realist evaluation, outcomes are not simply end-points but anticipated, intended, unintended or emergent changes that occur through the activation of mechanisms in particular contexts. It would therefore be helpful to specify what forms of impact are anticipated in this study, and whether these may include clinical outcomes, professional behavior, audit participation, data quality, quality improvement activity, governance, service development, policy influence, or some combination of these. Similarly, the IHFD as the intervention could be described a little more explicitly, including the particular audit components expected to contribute to these outcomes.
4. The candidate theories presented in Table 2 are useful starting points, but they remain broad at this stage. They may be strengthened by making the context, mechanism and outcome elements more visible, particularly the reasoning or responses triggered among stakeholders. This would help the reader to see how the proposed focus groups, expert panel input and other data sources will contribute to CMOC development and refinement.
5. Minor clarification of terminology may also be helpful. Table 2 is titled “Candidate theory examples”, but the theories are labelled as IPT 1 and IPT 2. It would be useful for the authors to clarify whether these are candidate theories, initial program theories, or early theory fragments intended to inform subsequent IPT development.
6. Some additional detail on how the proposed realist-informed focus group format, sampling across sites and professional groups, and the intended role of focus group data in refining candidate theories/IPTs and CMOCs is envisaged would also improve transparency.

These clarifications do not require changes to the study design, but would help readers understand how participant accounts, audit data and documentary evidence will contribute to program theory refinement.  In summary, this is worthwhile and important work.

Is the study design appropriate for the research question?

Yes

Is the rationale for, and objectives of, the study clearly described?

Partly

Are sufficient details of the methods provided to allow replication by others?

Partly

Are the datasets clearly presented in a useable and accessible format?

Not applicable

Reviewer Expertise:

My expertise is in realist review and realist evaluation, Lean and quality improvement in healthcare, person-centred process improvement, and the evaluation of complex healthcare interventions.

We confirm that we have read this submission and believe that we have an appropriate level of expertise to confirm that it is of an acceptable scientific standard, however we have significant reservations, as outlined above.

# Response

**Published:** 2026-08-18

**Authors:** Louise Brent

**Affiliations:** National Office of Clinical Au, Royal College of Surgeons in Ireland, Dublin, Leinster, Ireland

Thank you, Sean Paul and Anne Marie, for your review and really helpful feedback. 

 I have made the following amendments to the manuscript in response. 

- I have added more detail about the Irish Hip Fracture Database (IHFD) in the introduction including what constitutes 'impact' in relation to the study. 
- I have added an appendix to show the template used to extract data from papers. 
- In the methods section more detail was added to explain the realist logic underpinning this work and about how the initial program theories will be developed, tested and refined across the different phases of the study.
- The examples of programme theories in Table 2 were also updated to give examples of context, mechanisms and outcomes (CMO's) and I have also clarified that these are just examples of programme theories.
- Details were added to describe the focus groups in terms of sampling and composition, and the iterative process including literature search, research group, expert panel and study participants role were also expanded.  
- Changes to some references were made or added.

# Reviewer report: Reviewer response for version 1

**DOI:** 10.21956/hrbopenres.15925.r55829

**Published:** 2026-06-17

**Authors:** Lenore Anne de la Perrelle

**Affiliations:** 1 College of Nursing and Health Sciences, Flinders University, Bedford Park, Australia

This protocol sets out the rationale and steps in conducting a realist evaluation to identify how clinical audit generates impact, for whom and in what context. To improve the protocol to be more transparent and a realist evaluation, I suggest a few changes to the order and description.

 The *methodology* for a realist evaluation needs to be more clearly outlined, at the beginning rather than the *methods* that you lead with.  The methodology is theory articulation, the refinement and then the verification process, which should act as the framework for this study.  

  It would be useful to clarify how the audit intervention is meant to work in the IHFD, what resources are used and what the intended outcomes are. Impact is a general term and needs to be defined better.  A program logic would help to identify which specifics of the audit process matter in achieving outcomes as intended.

 In Table 1, two candidate theories are provided as examples, but the 'who' is not clear in the first one (who focuses on quality improvement and why? is this the outcome intended for the audit and why) It is not articulated as a candidate theory.  A suggested wording might encompass a hypothesis 'If Auditors/ healthcare professionals focus on quality improvement rather than assurance, they can make changes which satisfies their sense of professional competence'. look to identify what their rationale might be. You can label the parts of the if-then statements with C M O.

 Then describe the steps within each aspect of the framework more clearly in sequence such as how you will construct the ITPs (expert panel), how you search for literature to test them (scoping review, iterative searches) and how you will gather evidence to refine or verify the theories.

  The methods for gathering the evidence can be described further, such as the focus group discussions, surveys and scoping review.

 Examples of realist-style questions to be asked within the focus groups and expert panel would strengthen the claim to be a realist evaluation. The rationale for the participants' thinking needs to be identified and collected.

 Figure 1 needs to be made more relevant to your study and labelled "adapted" from Pawson and Tilly and Mukumbang et al, as it is not the same as produced by them.

Is the study design appropriate for the research question?

Yes

Is the rationale for, and objectives of, the study clearly described?

Partly

Are sufficient details of the methods provided to allow replication by others?

Partly

Are the datasets clearly presented in a useable and accessible format?

Not applicable

Reviewer Expertise:

My expertise is in realist synthesis and evaluation of QI in health care,  mixed methods and qualitative research in aged care, palliative care and trauma-informed care

I confirm that I have read this submission and believe that I have an appropriate level of expertise to confirm that it is of an acceptable scientific standard, however I have significant reservations, as outlined above.

# Response

**Published:** 2026-08-18

**Authors:** Louise Brent

**Affiliations:** National Office of Clinical Au, Royal College of Surgeons in Ireland, Dublin, Leinster, Ireland

Thank you, Lenore Anne, for your review and comments, they are really welcome and helpful.

 In response I have made the following edits to the manuscript. 

- I have outlined details about the framework used to describe realist evaluation methodology in the introduction section and also expanded on the audit intervention detail and described what is meant in terms of 'impact'. 
- I updated Table 2 describing two candidate theories to reflect your suggestion. I renamed these programme theory examples.
- I have described the steps within each aspect of the framework more clearly in sequence such as how you will construct the ITPs including details about the iterative nature of the study including the literature search, research team, expert panel and study participants.
- I also included an example of realist-style questions. 
- I amended the title for Figure 1 (now Figure 2) as you recommended. 
